# Quantification of the contribution of septal movement to stroke volume in healthy subjects, athletes, patients with pulmonary insufficiency and patients with pulmonary hypertension

**DOI:** 10.1186/1532-429X-14-S1-P87

**Published:** 2012-02-01

**Authors:** Sigurdur S Stephensen, Katarina Steding, Hakan Arheden, Einar Heiberg, Marcus Carlsson

**Affiliations:** 1 Department of Clinical Physiology, Skånes University Hospital, Lund, Sweden; 2 Department of Pediatric Cardiology, Skånes University Hospital, Lund, Sweden

## Background

In theory, movement of the septum contributes to left ventricular stroke volume (LVSV) if the movement is toward the left ventricle (LV) in systole. If the septum movement is toward the right ventricle (RV), as in dyssynchrony, the septal contribution to LVSV decreases. In patients with increased volume load of the RV the septum may move towards the RV and contribute to right ventricular stroke volume RVSV) at the expense of LVSV. The amount of contribution to stroke volume from septal movement is not kown.

The aim of the study was to quantify the percentage of the left ventricular stroke volume, that is derived by the septal movement during systole, in healthy volunteers and patients with volume or pressure load of the RV.

## Methods

Cardiac MRI was used for three-dimensional assessment of cardiac volumes and septal movement. Four groups were examined; healthy subjects (n=29), athletes with high aerobic capacity (n=12), patients with an increased right ventricular volume load from moderate or severe pulmonary insufficiency (PI) in surgically corrected tetralogy of Fallot (n=20) and surgically corrected pulmonary stenosis (PS) (n=2) and patients with an increased right ventricular pressure overload secondary to pulmonary hypertension (n=11). LVSV was calculated by delineation of the endocardium of the LV in diastole and systole. The border between the LV and RV, i.e. the epicardial contour of the septum was delineated in diastole and systole from the apex to the base. Thereby the three-dimensional volume, derived from septal movement, was quantified.

## Results

Septal movement contributed to 8.7±0.7% of stroke volume in healthy subjects and 7.5±0.6% in athletes (p=ns). However, in patients with PI because of surgically corrected tetralogy of Fallot or PS, the septum contributes to right ventricular stroke volume by 14.1±1.7% (p<0.001 compared to healthy subjects). In patients with pulmonary hypertension the contribution to LVSV was 2.5±2.5% (p<0.001 compared to healthy subjects) with a wide range (19% to -9%) of left ventricular stroke volume.

## Conclusions

Septal movement contributes to 9% of LVSV in healthy subjects. However, in volume overload of the RV, the septum contributes significantly to RVSV by 14%. In patients with pulmonary hypertension there is a wide variation in septal contribution to LVSV.

## Funding

No funding.

